# Legitimacy-building to establish climate services in Uganda’s health system: The role of digital health platform

**DOI:** 10.1016/j.ssmhs.2026.100212

**Published:** 2026-06

**Authors:** Patrick Okecho Omiel, Dueholm Müller Sune, Didacus Namanya, Prosper Behumbiize, Paul Mbaka

**Affiliations:** a Department of Informatics, HISP Centre, University of Oslo, Oslo, Norway; b Geographer & Focal Point (Climate Change and Health, and GIS), Ministry of Health, Kampala, Uganda; c HISP Uganda, Kampala, Uganda; d Division of Health Information Management, Ministry of Health, Kampala, Uganda

**Keywords:** Climate services for health, Digital health platforms, DHIS2, Legitimacy-building, Boundary objects

## Abstract

Establishing climate services within health systems is increasingly recognized as essential for strengthening resilience in low- and middle-income countries, yet little is known about how such efforts gain legitimacy. This case study examines the early-stage establishment of climate services within Uganda’s national health system through the DHIS2 digital health platform. Drawing on legitimacy theory and boundary object theory, we trace how actors mobilized pragmatic, moral, and emerging cognitive forms of legitimacy, and how the platform mediated these processes. We introduce the notion of digital health platforms as legitimacy infrastructure—socio-technical arrangements through which legitimacy is constructed, transferred, and normalized across institutional boundaries. The study identifies key legitimacy-building mechanisms, including co-creation practices that foster ownership, alignment with national policy priorities that secure institutional backing, and the use of trusted digital infrastructures that lower adoption barriers. By theorizing how platforms mediate cross-sectoral acceptance, the study contributes knowledge of why some innovations move from establishment toward institutionalization while others falter. For policymakers and implementers, this case-study underscores the importance of embedding co-creation, aligning innovations with national frameworks, and establishing cross-sector collaboration early in the establishment of climate services for health, particularly when anchored in existing health information infrastructures.

## 1 Introduction

Climate change presents an escalating threat to human health. The World Health Organization has described it as one of the greatest health challenges facing humanity, warning that it could reverse decades of progress in development, global health, and poverty reduction while exacerbating existing health inequities (WHO, 2008, 2023). In response, there have been growing calls for adaptation strategies that enable health systems to anticipate, prepare for, and respond to climate-related risks, including through the use of climate services in health decision-making (Corral et al., 2012; WHO, 2015).

Climate services for health are increasingly recognized as essential for strengthening health system resilience. They encompass collaborative processes through which climate knowledge is produced, tailored, communicated, and used to support public health decisions across different temporal and spatial scales (WMO, 2023). Rather than merely providing information, climate services aim to enable timely, localized, and operational decisions, such as supporting vector control campaigns, forecasting disease outbreaks, and prioritizing high-risk populations and areas (Fletcher et al., 2021; Thomson et al., 2019).

Despite their potential, the establishment of climate services within health systems remains uneven in many low- and middle-income countries (LMICs). Efforts are often constrained by fragmented institutional arrangements, limited technical and data capacity, and weak coordination across climate and health sectors (Dinku et al., 2022; Hussey and Arku, 2020). These challenges point to the need for information systems-based approaches that can embed new forms of data and analytic capability within routine health decision-making structures, particularly in resource-constrained settings facing rapidly evolving climate risks (Manyuchi et al., 2021, 2023)

In this context, digital health platforms have emerged as important infrastructures through which new data sources and analytic tools can be introduced into health systems. Digital health platforms such as DHIS2 (District Health Information Software 2)—an open-source health information system widely used across LMICs—offer a ready-made institutional and technical foundation for embedding climate-related data and tools into existing health information workflows (HISP Centre, 2024b). In Uganda, this potential is being explored through a pilot initiative in which climate data and analytic tools are being embedded within the national health information system to support climate-informed planning.

However, the success of such initiatives depends not only on technical feasibility but also on legitimacy, that is, the extent to which new data, tools, and practices are perceived as appropriate, desirable, and credible within existing institutional contexts (Suchman, 1995). Embedding climate services within a health information system represents an institutional innovation that challenges established roles, responsibilities, and decision-making practices, and therefore requires legitimacy to be actively constructed and sustained if it is to become normalized within the health system (Sahay et al., 2020; Scott, 2008).

This paper uses Uganda’s DHIS2 climate–health initiative as a case study to examine how legitimacy is built during the early stages of establishing climate services within a health system. It is guided by the following research question: **What actions do actors take to build legitimacy for the establishment of climate services in the health system, and how do digital health platforms mediate these actions?**

To address this question, the study draws on a dual theoretical lens that combines legitimacy theory with boundary object theory. Legitimacy theory provides a framework for understanding how new practices gain acceptance within institutional environments, while boundary object theory explains how shared socio-technical artifacts—such as digital health platforms—facilitate coordination and alignment. We conceptualize the establishment of climate services within the health system through the DHIS2 not as a purely technical exercise, but as an innovation that requires new forms of collaboration, interpretation, and justification across the climate and health sectors.

The remainder of the paper is structured as follows. Section 2 reviews the relevant literature. Section 3 presents the theoretical framework. Section 4 describes the research methods. Section 5 presents the findings, followed by the discussion in Section 6. Section 7 outlines the study’s limitations and directions for future research, and Section 8 concludes.

## 2 Related literature

Research on institutional innovation has long recognized legitimacy as a critical condition for the adoption, sustainability, and normalization of new practices, technologies, and institutional arrangements. Suchman’s (1995) typology distinguishing pragmatic, moral, and cognitive legitimacy remains central to understanding how new practices, technologies, or organizational forms become accepted within established institutional environments. Subsequent scholarship has applied this framework across diverse organizational and sectoral contexts, emphasizing that legitimacy is socially constructed and actively negotiated rather than automatically conferred (Suddaby et al., 2017). Despite its foundational role in institutional theory, however, the explicit application of legitimacy concepts to digital health systems particularly in LMICs remains limited.

Within digital health, legitimacy-building efforts have been shown to focus on three interrelated challenges: convincing stakeholders to trust the system, ensuring alignment with established institutional norms and governance arrangements, and demonstrating practical value through concrete use and outcomes (Greenhalgh et al., 2017). In LMIC settings, these challenges are often intensified by resource constraints, fragmented governance, and competing sectoral priorities (Kiberu et al., 2014). Evidence from health information systems implemented through platforms such as DHIS2 highlights that legitimacy is not given but negotiated through ongoing interaction between global design standards and local institutional practices, requiring both technical adaptation and institutional alignment (Braa and Sahay, 2012; Sahay et al., 2020). Empirical applications of legitimacy theory to DHIS2 remain rare; for example, (Msendema et al., 2024) show how legitimacy for data quality management practices in Malawi was constructed through isomorphic mechanisms. Such analyses, however, remain scarce and are almost entirely absent in the specific domain of climate–health initiatives.

When institutional innovations in cross-sectoral boundaries—as in efforts to establish climate services within health systems—legitimacy-building becomes more complex. These initiatives must satisfy multiple institutional logics, evaluation criteria, and accountability structures while maintaining coherence across diverse organizational contexts (Bryson et al., 2015). Cross-sectoral collaboration literature highlights how such settings are characterized by coordination challenges, role ambiguity, and contested authority. In this context, boundary object theory offers a useful lens for understanding how coordination can be achieved across heterogeneous communities of practice.

Originating with (Star and Griesemer, 1989), boundary object theory explains how collaboration is enabled among actors with distinct interests, expertise, and interpretive frames through shared artifacts that are “plastic enough to adapt to local needs… yet robust enough to maintain a common identity” (p. 393). In digital health, platforms increasingly function as such boundary objects, accommodating multiple interpretations and uses while retaining a stable technical architecture and shared functional purpose that ensures coherence across stakeholders. Empirical studies illustrate this dynamic. Personal health record systems, for example, allow clinicians, patients, and administrators to engage with the same artifact while extracting different forms of value—clinical, personal, and administrative respectively (George and Kohnke, 2018). Similarly, video-supported digital toolkits have demonstrated how flexibility enables adaptation to local professional practices, while standardization provides a shared reference point for cross-boundary collaboration (Saidi et al., 2023). In these cases, digital systems function as boundary objects by accommodating diverse perspectives while maintaining coherence across professional groups.

Recent scholarship has begun to explicitly link boundary object theory to legitimacy-building processes. This work shows that the interpretive flexibility of boundary objects allows different stakeholder groups to align innovations with their own priorities, values, and practices, thereby supporting pragmatic legitimacy through perceived usefulness, moral legitimacy through alignment with normative goals, and cognitive legitimacy by embedding new practices within familiar frameworks (Do et al., 2024). While legitimacy theory clarifies how innovations are evaluated and accepted, it offers limited insight into the socio-technical mechanisms through which coordination across sectoral boundaries occurs. Conversely, boundary object theory illuminates mechanisms of cross-boundary collaboration—such as translation across communities of practice and coordination without full consensus—but does not fully explain how these mechanisms translate into institutional acceptance. Their complementary use therefore provides a more comprehensive understanding of how cross sectoral innovations are both coordinated and legitimated.

In parallel, climate change is increasingly recognized as a public health emergency, yet the establishment of climate services within health systems remains uneven, particularly in LMICs. Although conceptual frameworks and implementation plans for climate services for health are well developed (WHO-WMO, 2019, 2023), their operationalization has proven challenging due to sectoral silos, data interoperability constraints, and unclear institutional mandates (Barteit et al., 2023; Dimitrova et al., 2023). Digital health platforms such as DHIS2 offer a potentially powerful means of addressing these challenges. Their widespread use within national health information systems positions them as promising infrastructures for embedding climate-related data and analytic tools into routine health system practice (HISP Centre, 2024a). However, existing literature provides limited insight into how such platforms mediate legitimacy-related challenges associated with introducing new forms of data and knowledge into health systems.

Recent work has examined legitimacy challenges faced by digital platforms engaging with established sectors. (Essen et al., 2023) show that, for commercial platforms entering domains such as healthcare, legitimacy is contested, negotiated, and gradually stabilized, co-evolving with platform business models during early phases of entry. However, this work focuses on market-oriented platforms in high-income contexts. In contrast, public-sector digital health platforms in LMICs are often already institutionalized within state governance arrangements, shifting the legitimacy challenge from platform entry to how new data sources, tools, and practices gain acceptance when embedded in trusted infrastructures. Platforms such as DHIS2 therefore sit at the intersection of legitimacy and boundary object theory: as institutionalized health information infrastructures, they combine interpretive flexibility and structural coherence with accumulated legitimacy from established practices, enabling them to function as legitimacy sponsors (Sahay et al., 2020) for newly embedded innovations—particularly in cross-sectoral initiatives such as climate services for health.

Despite these conceptual complementarities, few empirical studies have explicitly integrated legitimacy theory and boundary object theory to analyze digital platforms in public-sector health systems. While legitimacy-focused work exists in digital health (Msendema et al., 2024), and boundary object theory has been used to study how digital tools enable collaboration across professional domains (Popper, 2015; Saidi et al., 2023), such applications remain fragmented. Moreover, little is known about how platforms mediate legitimacy-building during the early stages of institutional innovation—before new practices become routine or taken-for-granted. Addressing this gap, the present study examines how the DHIS2 digital platform mediates early-stage legitimacy-building for the establishment of climate services within Uganda’s health system, advancing understanding of how such platforms support cross-sectoral coordination and institutional acceptance of emerging practices in resource-constrained settings.

## 3 Theoretical framework

This study draws on a dual theoretical lens that combines Suchman’s (1995) legitimacy theory with boundary object theory (Star and Griesemer, 1989) to examine how actors build acceptance for establishing climate services into health systems through digital platform capabilities. As alluded, legitimacy theory offers a means to understand what actions were taken by actors and why it matters, while boundary object theory explains how digital platforms facilitate the process of legitimacy-building.

Legitimacy, as defined by Suchman (1995), is “a generalized perception or assumption that the actions of an entity are desirable, proper, or appropriate within some socially constructed system of norms, values, beliefs, and definitions” (p. 574). It is not an intrinsic attribute of an innovation but a social judgment that must be actively cultivated, particularly when innovation challenges established routines or practices. Suchman distinguishes between pragmatic legitimacy, which is based on stakeholders’ perception that an innovation serves their immediate interests, either through direct benefits (*exchange*), responsiveness to broader constituent concerns *(influence)*; moral legitimacy, which rests on normative evaluations that the innovation is the “right thing to do,” whether because its *outcomes* are beneficial (*consequential*), its *procedures* are fair and transparent *(procedural)*, or its *structures* and roles are appropriate *(structural)*; and cognitive legitimacy, which derives from the innovation being comprehensible (*comprehensibility*) and eventually accepted as an inevitable, taken-for-granted feature of the environment *(taken-for-grantedness)*. These categories provide a multi-dimensional framework for analyzing how new or contested practices gain institutional acceptance.

Boundary object theory complements this by explaining how artifacts facilitate coordination between communities of practice with different perspectives, interests, and vocabularies (Star and Griesemer, 1989). Boundary objects are “plastic enough to adapt to local needs… yet robust enough to maintain a common identity” (p. 393), allowing actors to engage with them differently while still enabling collective action. In a digital platform context, boundary objects possess *interpretive flexibility*, allowing multiple, context-specific meanings; *structure coherence* provisioning standardized forms enabling coordination, and a *common identity*, ensuring consistency across different settings (Fleming et al., 2023).

To further specify actor roles in legitimacy-building, we draw on (Hussain et al., 2004) distinction between legitimacy seekers (actors promoting climate service establishment via DHIS2), legitimacy providers (actors with authority to confer acceptance), and dual-role actors who both seek and provide legitimacy. These actor groups engage with DHIS2 differently: seekers use the platform to demonstrate value and alignment; providers interpret this effort through established institutional frameworks; and dual-role actors bridge perspectives across their multiple affiliations.

Bringing these elements together, the framework directs attention to how the DHIS2 digital health platform enables and mediates actor actions through which legitimacy is constructed. Rather than treating legitimacy as a static outcome, we examine how the platform supports actors in establishing climate services by demonstrating their instrumental value (pragmatic legitimacy), normative appropriateness (moral legitimacy), and increasing taken-for-grantedness within routine health system operations (emerging cognitive legitimacy).

## 4 Methods

This study forms part of an ongoing qualitative longitudinal case study of climate–health initiatives in Uganda, conducted at national and district levels since April 2024 as part of the DHIS2 Climate–Health Project (HISP Centre, 2024a). The analysis focuses on the early-stage establishment of climate services, examining initial efforts to build acceptance rather than longer-term processes of institutionalization. Empirically, the study investigates legitimacy-building mechanisms within and across Uganda’s health and meteorological sectors, including interactions among technical teams, governance structures, and implementation partners.

An interpretivist case study methodology was adopted (Klein and Myers, 1999), enabling close engagement with actors’ meanings, practices, and interpretations as legitimacy was actively constructed for establishing climate services in the health sector. The research data were generated through qualitative methods including participant observations, document and system reviews (Orlikowski, 2000), allowing us to trace actor actions and role of the DHIS2 platform.

### 4.1 Study setting and context

Uganda has established a comprehensive policy and institutional framework for climate change adaptation, including the National Climate Change Policy (2015), the Climate Change Act (2021), and the Health National Adaptation Plan (H-NAP), which guides climate adaptation within the health sector (MOH Uganda, 2024). Climate governance is coordinated by the Ministry of Water and Environment, which hosts the national meteorological services, while the Ministry of Health (MOH) leads climate change and health activities supported by established technical working group structures. This setup is characterized by institutional separation alongside operational interdependence, shaping how climate services for health can be authorized, coordinated, and enacted across sectoral boundaries.

For the health sector, the health information system is decentralized and anchored in DHIS2, as a mandated platform for routine health data management. Implemented since 2010, DHIS2 is now deeply embedded in health sector practice, supporting surveillance, reporting, planning, and program monitoring across administrative levels (Kiberu et al., 2014). As articulated in Uganda's adaptation plan, DHIS2 is envisaged as the primary interface through which climate-related data and analytics can support climate-informed decision-making within the health system.

Despite this strategic vision, climate service inclusion into routine health information practices remains limited, with access to climate–health information often relying on ad hoc coordination between health and meteorological institutions. This reflects both the relative infancy of the climate–health domain and the challenges of aligning data, tools, and mandates across sectors with distinct institutional logics. Consequently, establishing climate services for health requires not only technical integration but also the negotiation of roles, responsibilities, and legitimacy.

In response, and following the emergence of DHIS2 climate tools, the health ministry, in collaboration with its HISP Uganda, a longstanding digital health partner, initiated a climate–health pilot in 2024 to include climate tools into the national health information system as a pathway for establishing climate services for health. As an early-stage initiative anchored on already trusted digital infrastructure, the pilot provides an information-rich case for examining how legitimacy for climate services is constructed and extended through routine health system platforms.

### 4.2 Data collection

As summarized in Table 1, data collection drew on three complementary sources: policy and governance documents, project and system artefacts, and participant observation. Together, these sources enabled triangulation of institutional framing, platform properties, and legitimacy-building practices during the early establishment of climate services for health.

First, a targeted review of policy and governance documents provided the institutional and regulatory context shaping climate–health establishment. These documents included national adaptation policies, governance guidelines, and formal collaboration frameworks, which were analyzed to identify how climate services were framed in relation to national priorities, mandates, and sectoral responsibilities.

Second, project and system artefacts were examined to capture the technical and design dimensions of climate data integration. These artefacts included pilot implementation plans and reports, DHIS2 configurations, and tools such as the DHIS2 Climate App and the Climate and Health Analytics Platform (CHAP). Analysis focused on how climate data and analytics were configured, represented, and embedded within existing DHIS2 workflows, and on how the platform enabled the process of building legitimacy.

Lastly, participant observation constituted the primary empirical data source. The first author was embedded within the HISP Uganda implementation team and participated in routine project activities while maintaining an interpretive research stance. Observations were conducted across a range of settings, including health program meetings, technical working groups and subcommittees, health–meteorology engagement meetings, national stakeholder meetings, platform implementer meetings, DHIS2 configuration sessions, district-level meetings, and webinars. These observations captured real-time interactions through which legitimacy was sought, negotiated, and interpreted, including co-creation practices, governance processes, cross-sector coordination, and early routine use of climate tools.

### 4.3 Data analysis

Data analysis followed a qualitative, theory-informed coding approach using a deductive strategy (Fereday and Muir-Cochrane, 2006). Coding was guided by Suchman’s (1995) legitimacy typology; pragmatic, moral, and cognitive which provided an initial analytical framework for examining how actor actions contributed to legitimacy-building during the early establishment of climate services within the health system.

Analysis proceeded in two iterative stages. First, we identified and coded legitimacy-building actions across observational data, project documents, and field notes. These actions captured how actors articulated value, justified relevance, and sought acceptance for climate services in different organizational and governance settings. Each action was coded with attention to the actors involved and the situational context, allowing patterns of legitimation to be traced across sites and over time. As part of this stage, actors were categorized drawing on (Hussain et al., 2004) as legitimacy seekers, legitimacy providers, or dual-role actors, reflecting their differing capacities to pursue or confer legitimacy (see Table 2).

Lastly, we conducted focused coding of the mediating role of DHIS2, drawing on boundary object theory (Star and Griesemer, 1989). This analysis examined how the platform’s interpretive flexibility, structural coherence, and institutional embeddedness shaped cross-sector coordination and supported shared understandings of climate services across health and meteorological domains. Attention was directed to how DHIS2 structured interactions, stabilized meanings, and enabled legitimacy-building practices across diverse actor groups. This approach enabled us to examine how legitimacy was actively constructed through platform-mediated actions, and how DHIS2 shaped the conditions under which climate services became perceived as useful, appropriate, and routine. The resulting analytical synthesis is presented in Table 3 under the finding section.

## 5 Findings

The findings examine how legitimacy for climate services was constructed through concrete actor actions during the early-stage establishment process. Drawing on Suchman’s (1995) typology of pragmatic, moral, and cognitive legitimacy, the analysis highlights the actors involved and the ways in which DHIS2 mediated these processes as a boundary object enabling coordination across institutional boundaries. Table 3 summarizes the key legitimacy-building actions, actors, and platform roles identified in the analysis.

### 5.1 Building pragmatic legitimacy through alignment and demonstrated value

Pragmatic legitimacy was primarily constructed through deliberate alignment with nationally recognized priorities and by demonstrating the feasibility and instrumental value of climate services for health programs and research. Central to this effort was framing the establishment of climate services as a direct contribution to Uganda’s adaptation, which explicitly identifies climate services for health as a core adaptation strategy. As stated in the plan *“Climate services for health represent a crucial component in addressing climate change issues in Uganda […] The meteorological data generated by UNMA should be integrated with health data from DHIS2 for early warning and early action”* (MOH Uganda, 2024, p. 16).

Building on this explicit strategic mandate, platform implementers and health system focal units for climate change and health actively reinforced this alignment across multiple fora, including technical working groups, senior management meetings, and public engagements. In these settings, DHIS2 was consistently positioned as a practical mechanism for operationalizing national adaptation commitments, rather than as an isolated or experimental pilot. This framing was particularly effective in engaging legitimacy providers—health programs, technical working groups, sector leaders, and climate data authorities—who interpreted the initiative as both policy-aligned and institutionally appropriate. As remarked during a national climate–health forum: *“DHIS2 is a key component of the H-NAP because it allows us to integrate climate data with health data already managed within the system. When you talk about the role of DHIS2, it is essential*—*it will help us develop early warning systems to tackle diseases like cholera, which are very common in Uganda. This makes it a very useful tool for implementing the plan*.*”*

Through such framing, the climate service establishment was positioned not as an actor-driven innovation but as a necessary step toward implementing nationally endorsed objectives. DHIS2’s interpretive flexibility played a key mediating role in this process, allowing the same platform to resonate across different audiences: policy actors viewed DHIS2 as an adaptation instrument, health program officers engaged with it as an extension of routine analytics and surveillance, and climate authorities interpreted it as a legitimate interface for operational data use. In this way, abstract policy commitments were rendered actionable through dashboards and analytics that enabled cross-actor recognition of value.

**Fig. 1:**
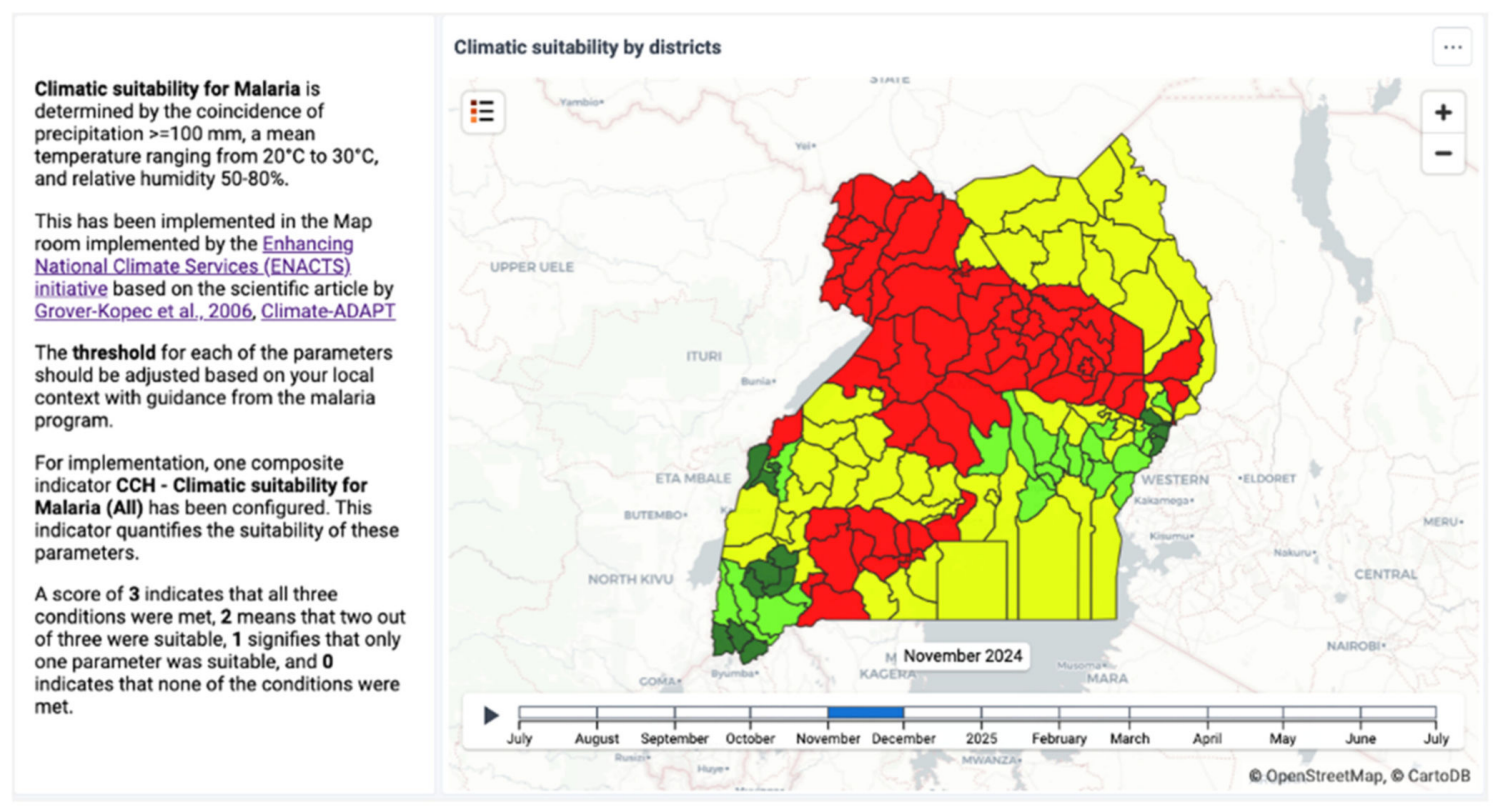
Sample dashboard showing monthly climatic suitability for malaria by district.

Pragmatic legitimacy was further strengthened through exchange-based mechanisms as actors experienced direct, program-relevant benefits from the climate services. Through a co-creation process, platform implementers and health system focal units worked with national programs—including malaria, nutrition, and non-communicable diseases—to develop program-specific climate–health use cases within DHIS2. These efforts enabled health programs, researchers, and subnational users to engage with tailored analytics that addressed concrete planning and research needs. As illustrated in Fig. 1, the malaria climatic suitability analysis embedded within DHIS2 supported improved stratification of climate-risk districts and more targeted intervention planning. Reflecting on this value after a demonstration during a partner meeting, a malaria program officer noted: *“We have been looking forward to this climate data and analysis to integrate into our planning of interventions. Now, we have something that we can work with for the program. Let us all embrace this and work with the team to make improvements as necessary*.*”*

Here, DHIS2’s ability to support tailored analytics within a unified national platform was central. By enabling diverse users to extract program-specific value without fragmenting system identity, the platform sustained interpretive flexibility while maintaining a shared analytical and governance framework. Beyond programmatic use, researchers expressed strong interest in accessing harmonized climate–health datasets through formal health ministry channels, while climate indicators were incorporated into Uganda’s malaria scorecard submitted to regional accountability mechanisms. Together, these practices reinforced pragmatic legitimacy by demonstrating that climate services embedded within DHIS2 delivered tangible benefits across multiple constituencies and institutional levels.

### 5.2 Moral legitimacy through procedural compliance and ethical justification

Moral legitimacy was constructed by demonstrating that the initiative adhered to established institutional procedures and advanced ethically valued public health goals. A first pathway involved securing formal approval and ownership through the health ministry governance processes. Platform implementers, working closely with health system focal points, engaged established technical working groups, subcommittees, and senior management forums to present the initiative, demonstrate its scope, and clarify governance arrangements. Early endorsements were often conditional, reflecting expectations of procedural rigor rather than uncritical support. As one technical working group member noted during deliberations: *“From my side, this is one of the things that we have been talking about, multisectoral integration to address social and environmental determinants. This is one good example that is very welcome, I have no objection […] As we endorse this innovation for piloting, we will need a detailed concept of the project stipulating the timeline clearly, stating who is doing what and when*.*”* These interactions positioned health programs, technical working groups, and sector leaders as legitimacy providers, whose approval depended on adherence to recognized procedures and accountability norms. Over time, health system focal units—initially operating as intermediaries—transitioned into more active leadership roles, presenting the initiative in senior management meetings and advocating for its progression within institutional forums. This shift signaled growing internal ownership and trust, marking a movement from conditional endorsement toward moral acceptance grounded in procedural compliance.

DHIS2 played a critical role in this process by generating standardized outputs—dashboards, indicators, and analytics—aligned with existing reporting structures and review practices. This structural coherence allowed climate services to be evaluated using familiar criteria, reinforcing institutional fit and procedural credibility. Rather than introducing new or parallel decision processes, the platform enabled climate information to circulate through established governance channels, supporting moral legitimacy based on appropriate procedures and organizational arrangements.

A second pathway to moral legitimacy involved the establishment of a formal collaboration framework between the health and meteorological service. Initially, skepticism within meteorology reflected concerns that the initiative was a partner-driven research activity rather than a government-led service. This perception shifted as platform implementers and health system focal points reframed the initiative as aligned with national mandates and formalized through an inter-agency agreement. A senior meteorological officer described this change in perspective: *“With the climate crisis, it feels as though we are all on the same plane. If something goes wrong, we all go down together. While we are still in these positions, we must take actions that will have lasting impact for generations to come. Having this agreement written down makes sense; it serves as proof of our commitment to the health sector*.*”*

The collaboration framework clarified institutional roles, established reciprocal data-sharing arrangements, and formalized cross-sector responsibilities. In this context, DHIS2 served as a neutral coordination space with a recognized common identity, reducing contestation over authority and ownership. Because the platform was already institutionally anchored within the health system yet technically accessible to meteorological authorities, it enabled cooperation without requiring either sector to relinquish mandate or control.

Consequential moral legitimacy was further reinforced by framing the initiative as ethically justified due to its anticipated societal benefits. Across stakeholder groups, actors emphasized the role of climate services in strengthening early warning systems, improving preparedness for climate-sensitive diseases, and protecting vulnerable populations. By demonstrating climate service with standardized indicators embedded within routine reporting and planning workflows, DHIS2 platform rendered these ethical benefits visible in forms already recognized as authoritative and appropriate within the health system. Together, procedural compliance and ethical justification reinforced moral legitimacy by demonstrating that establishing climate service was not only useful, but institutionally proper and normatively aligned with public health responsibilities.

### 5.3 Emerging cognitive legitimacy through operational fit and routine use

**Fig. 2:**
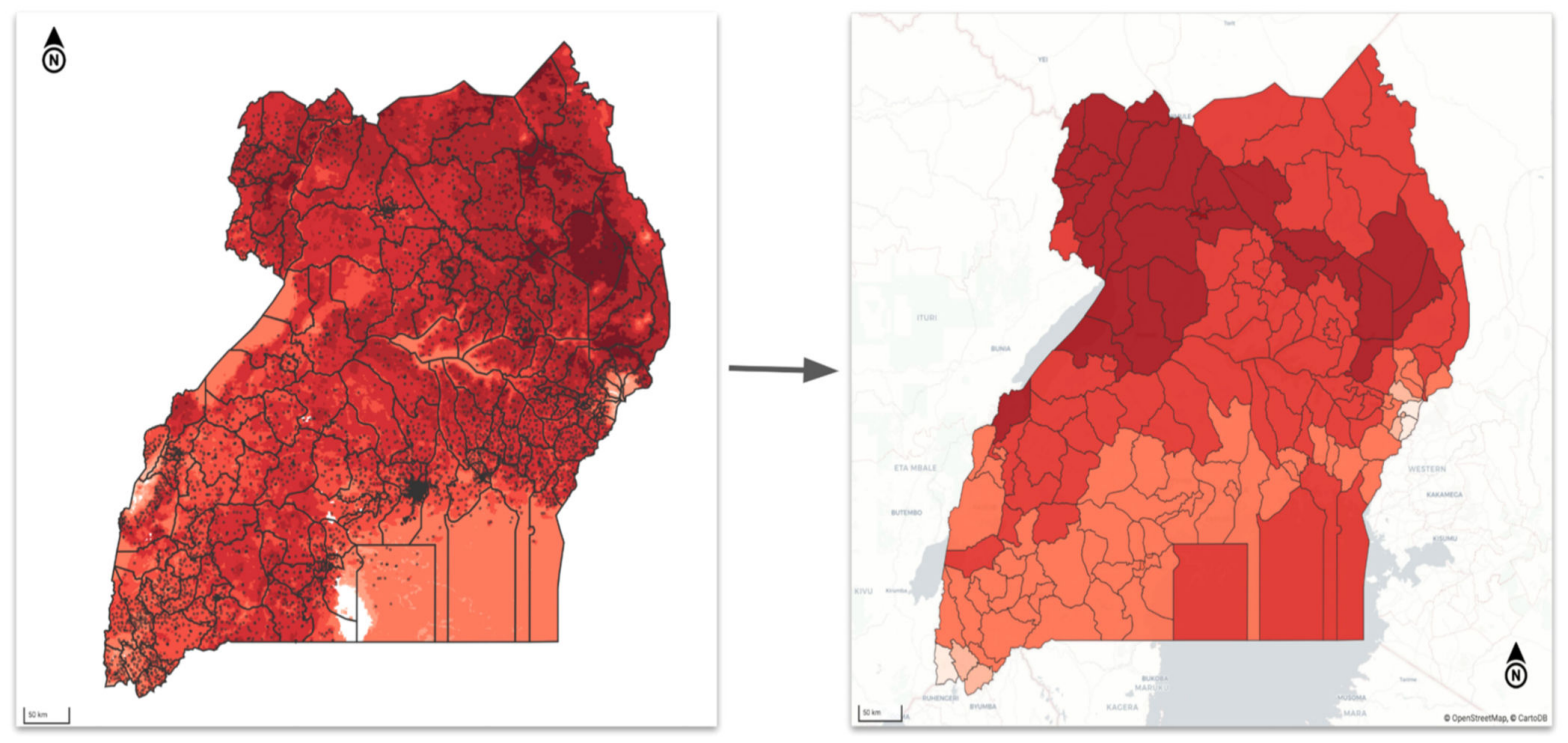
Spatial alignment of climate data: gridded data into polygon formats to match health data structures.

Emerging cognitive legitimacy became visible as climate services were deployed into routine DHIS2 workflows and supported for early use. Platform implementers and health system focal units deliberately embedded climate tools within familiar interfaces, reporting periods, and spatial hierarchies (see Fig. 2), allowing users to engage with climate data without having to learn a new system. This operational fit supported comprehensibility, as climate information was presented through the same analytic structures already used for routine health data. As a health data officer explained during a coordination meeting: *“All that we need is for the climate data to be in the DHIS2 system like other health data; then we should be able to do our analysis as usual, comfortably […] we can easily generate pilot tables and even export the climate and health data for further analysis as we wish”*

By embedding new climate functions into existing DHIS2 periods, hierarchies, and analytics, the platform lowered cognitive barriers and reduced the sense that climate services represented a separate or unfamiliar practice. DHIS2’s structural coherence enabled climate data to be interpreted through established routines of reporting and analysis, allowing sub-national users, external partners, and IT support teams to recognize the tools as consistent with existing health information practices rather than as experimental add-ons.

Early signs of taken-for-grantedness began to emerge following the deployment of climate tools into Uganda’s DHIS2 production environment. District health teams were introduced to the tools through national webinars and subsequently began incorporating climate–health into their data review meetings and planning discussions. Reflecting on this shift, a Sub-national user noted: *“For a long time, we have wanted access to climate data for our district planning, but it has always been difficult to obtain. Now that we have access, especially through DHIS2, this will help us start including it in our analysis”*

While still at an early stage, these practices indicate a transition from experimental engagement toward routine use. In participants’ accounts, DHIS2’s common identity as Uganda’s mandated national health information system played a decisive mediating role: once climate data and tools were embedded in DHIS2, they were understood as part of legitimate health system practice. In this way, the platform functioned not only as a technical enabler but as an institutional support structure that anchored new climate–health capabilities within established norms and routines, enabling early steps toward normalization.

**Fig. 3:**
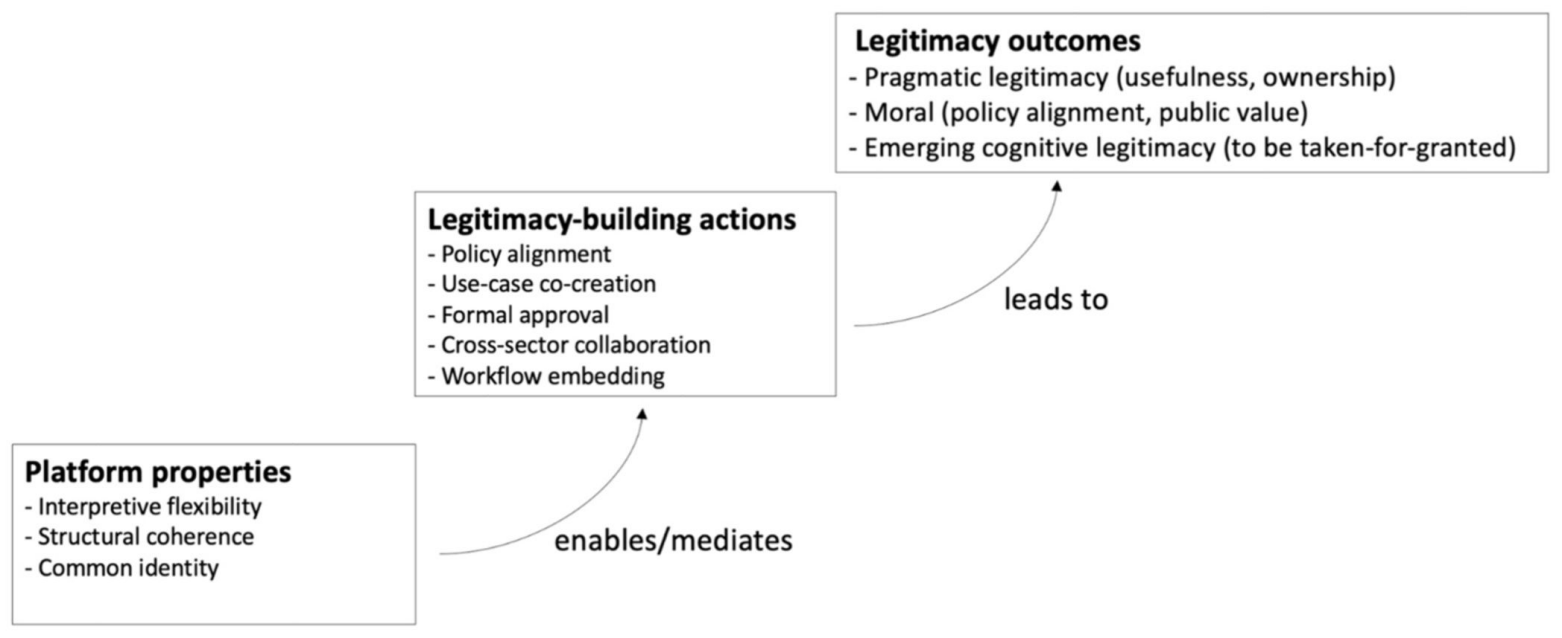
Linking platform properties, legitimacy-building actions, and legitimacy outcomes.

Overall, the findings show that legitimacy for establishing climate services emerged through a sequence of interconnected actor actions, including policy alignment, use-case co-creation, formal approval, cross-sector collaboration, and workflow embedding. As summarized in Table 3, these actions collectively contributed to pragmatic, moral, and emerging cognitive legitimacy during the early establishment phase. Fig. 3 synthesizes these findings by illustrating how the properties of the digital platform enabled and mediated the observed legitimacy-building actions, and how these actions contributed to different forms of legitimacy. Through this interaction, the platform supported coordination across institutional boundaries and translated actor efforts into broader acceptance, laying the groundwork for the normalization of climate services within routine health system practice.

## 6 Discussions

Drawing on Suchman’s legitimacy framework and boundary object theory, this study shows how legitimacy for climate services is actively constructed through socio-technical processes mediated by a digital health platform. The findings demonstrate how strategic actions and the properties of DHIS2 shaped the emergence of legitimacy during the early establishment of climate services within Uganda’s health system. In this section, we discuss the theoretical and practical implications of these findings. We first reflect on how digital platforms function as boundary objects that mediate acceptance across institutional domains, and extend this discussion by conceptualizing platforms as legitimacy infrastructure that anchors new innovations within trusted systems. We then consider what these insights imply for the design, governance, and scaling of climate services for health in LMIC contexts.

### 6.1 Theoretical implications

This study contributes to legitimacy and digital platform scholarship by showing how boundary objects function as legitimacy-building mechanisms in institutional innovation, particularly when new practices are embedded within already institutionalized digital platforms. While Suchman’s (1995) legitimacy framework conceptualizes legitimacy as pragmatic, moral, and cognitive acceptance, it has largely been applied at the organizational level. Our findings extend this perspective by demonstrating how material artefacts; specifically digital platforms, mediate legitimacy building across institutional boundaries, where acceptance must be negotiated among multiple sectors and authorities.

In doing so, the study aligns with and extends (Johnson et al., 2006) view of legitimacy as a dynamic social process unfolding through innovation, local validation, diffusion, and general validation of a new idea, practice, etc. In the Ugandan case, climate service establishment followed this trajectory: co-created use-cases constituted an institutional innovation; their embedding within program workflows reflected local validation; cross-sectoral uptake through technical working groups supported diffusion; and DHIS2’s institutional anchoring signaled early steps toward general validation. By shifting the analytical focus from organizations to institutional arrangements, the study advances legitimacy theory to better account for innovations that require coordination among diverse actors and governance regimes across organizational boundaries.

Building on prior work that conceptualizes boundary objects as “legitimacy vehicles” (Do et al., 2024), this study further specifies how the core characteristics of boundary objects support distinct forms of legitimacy. Interpretive flexibility allows actors to frame the establishment of climate service in ways that align with their institutional interests, fostering pragmatic legitimacy. Structural coherence embeds shared values and ethical commitments within standardized procedures, reinforcing moral legitimacy. Common identity anchors new practices within familiar cognitive frames, facilitating early normalization and contributing to cognitive legitimacy. This mapping between boundary object characteristics and legitimacy types represents a novel theoretical contribution, demonstrating the complementarity of these perspectives and foregrounding legitimacy-building as a socio-technical process. By explicitly linking boundary object characteristics to distinct forms of legitimacy, this study extends prior research that has primarily conceptualized boundary objects as mechanisms of coordination and translation (Carlile, 2002; Levina and Vaast, 2005; Star and Griesemer, 1989) and legitimacy as a perceptual evaluation process (Suchman, 1995; Suddaby et al., 2017), demonstrating how socio-technical artefacts actively participate in constructing institutional acceptance.

Beyond boundary objects, our findings reconceptualize digital platforms as legitimacy infrastructure. While prior studies recognize platforms as boundary objects (Terlouw et al., 2022), we introduce the notion of infrastructural anchoring: the process through which legitimacy of an established platform is conferred upon new innovations embedded within it. In Uganda, DHIS2’s status as the mandated national health information system meant that embedding climate services within the platform conferred implicit legitimacy; if it is part of DHIS2, it is understood as part of legitimate health system practices.

This insight extends emerging work that frames legitimacy as social infrastructure (Howe et al., 2025) by demonstrating that digital platforms themselves can provide such infrastructure, particularly in contexts characterized by institutional fragmentation or weak cross-sector coordination (Ahuja et al., 2023). In these settings, platforms function as institutional bridges, offering neutral spaces where innovations can gain legitimacy without requiring wholesale restructuring of mandates or authority.

Finally, the study advances digital platform scholarship by showing how platforms sponsor institutional innovations through their embeddedness in established governance structures, workflows, and mandates (Rao et al., 2008). We identify three interrelated mechanisms through which this sponsorship occurs: (1) legitimacy transfer, as credibility flows from the platform’s established role to new practices; (2) legitimacy translation, as new practices are embedded within existing routines and standards; and (3) legitimacy amplification, as platforms extend recognition across organizational and sectoral boundaries. Together, these mechanisms deepen our understanding of how digital platforms support the institutionalization of cross-sectoral innovations beyond their technical functions.

### 6.2 Practical implications

Our findings highlight co-creation as a critical strategy for establishing climate services that are both operationally relevant and institutionally accepted. Involving health program officers, meteorology officers, systems developers, and platform implementers in the co-development of climate–health use cases enhanced use, trust, and ownership. Prior studies similarly emphasize the importance of participatory approaches in climate services and digital health, showing that stakeholder diversity improves problem definition and supports sustainable adoption (Berkowitz, 2022; Le Minh et al., 2022; Stewart-Ibarra et al., 2022; Vandekerckhove et al., 2023). Our findings extend this literature by demonstrating that co-creation functions not only as a design principle but also as a legitimacy-building strategy. Participatory workshops and iterative prototyping allowed actors to articulate program-specific needs, fostering pragmatic legitimacy by demonstrating immediate value. At the same time, embedding responsiveness and inclusivity into the process reinforced moral legitimacy, while program-tailored dashboards normalized new practices within existing workflows, supporting emerging cognitive legitimacy.

Embedding climate tools within DHIS2 reduced adoption barriers by aligning new functionalities with established workflows and governance arrangements. While prior research highlights the sustainability advantages of government-owned platforms in LMICs (Msendema et al., 2024; Twabi et al., 2022; WHO, 2010; Woldeyohannes et al., 2023) our findings demonstrate an additional institutional effect: platform embedding enabled climate services to function as boundary objects that generated legitimacy. In this way, existing digital infrastructures provided not only technical capacity but also the institutional conditions necessary for sustained acceptance of climate service for health.

Explicit alignment with national policy frameworks, particularly Uganda’s adaptation plan, anchored climate service for health in recognized state priorities and extended legitimacy beyond partner-driven agendas. Extent research shows that policy coherence—especially through national adaptation plans—strengthens the implementation of climate initiatives (Paz-Soldán et al., 2023), while WHO’s and WMO’s joint guidance underscores policy alignment as foundational for institutionalizing climate services for health (WHO-WMO, 2019). Our findings demonstrate that such alignment is not automatic but requires deliberate negotiation across ministries, technical departments, and partners with distinct mandates. Framing the establishment of climate services in ways that resonate with existing health sector goals, demonstrating added value to health programs, and securing endorsement through technical working groups proved critical for institutional traction. Beyond describing these practices, our findings specify how such actions constitute legitimacy work within digital platforms, illustrating how acceptance of new innovations like climate service establishment is actively constructed rather than automatically conferred by technical efforts.

Finally, formalizing cross-sector collaboration through a data-sharing framework between the health-meteorology sectors helped create shared ownership and reduce inter-institutional tensions. Existing guidance highlights the importance of formal agreements, such as memoranda of understanding and joint technical working groups, for sustaining climate–health collaboration (Manyuchi et al., 2021; WHO-WMO, 2019). Our findings extend this guidance by showing that digital platforms can serve as neutral coordination spaces within such arrangements.

Platforms like DHIS2 provided a shared technical environment—through dashboards, indicators, and datasets—that anchored collaboration in data and workflows rather than sectoral hierarchies. By shifting interaction from mandate-based authority to jointly interpreted evidence, the platform reduced concerns over jurisdictional overreach and enabled evidence-driven dialogue and joint problem-solving. In this way, digital platforms function not only as information infrastructures but as institutional mediators that help stabilize cross-sector legitimacy.

## 7 Limitation and future research

This study has several limitations that point to avenues for future research. First, it focuses on early-stage legitimacy-building during a pilot phase in Uganda. While this offers rich insights into initial legitimation efforts, it does not capture longer-term processes of maintaining, repairing, or losing legitimacy as innovations scale and institutional contexts evolve. Future research should adopt longitudinal designs to examine how legitimacy dynamics change over time, particularly as digital platforms move from experimentation to routine use and as policy, organizational, and technological conditions shift.

Second, the analysis centers on health and meteorology sectors, yet similar legitimacy challenges are likely to arise in adjacent domains such as health–agriculture and health–environment integration, which are central to climate resilience. These domains intersect with climate–health in practice—for example, agricultural adaptation policies shape nutrition risk framing, while environmental governance influences the acceptance of climate data in health planning. Comparative and cross-sectoral studies could therefore extend understanding of how digital platforms mediate legitimacy across overlapping institutional fields, rather than within single sectoral interfaces.

Finally, the study gives limited attention to unintended consequences of platform-mediated legitimation. Critical ICT4D scholarship cautions that digital platforms may privilege certain actors, data sources, and forms of expertise while marginalizing others (Avgerou, 2017; Heeks et al., 2014). In our case, reliance on platforms such as DHIS2 may reinforce central-level authority at the expense of district priorities, or privilege global climate datasets over local meteorological or community knowledge. Future research should therefore examine not only how platforms enable legitimacy-building, but also how they shape power relations and knowledge hierarchies, and whose legitimacy is strengthened—or weakened—in the process.

## 8 Conclusion

This study examines how legitimacy was constructed during the early-stage establishment of climate services within Uganda’s health information system, showing how DHIS2 mediated pragmatic, moral, and emerging cognitive forms of legitimacy. By integrating legitimacy theory with boundary object theory, we advance the concept of digital platforms as legitimacy infrastructure—socio-technical arrangements through which legitimacy is constructed, transferred, and normalized across institutional boundaries.

Our findings demonstrate that legitimacy-building is not only driven by strategic actions, but also by the material and institutional properties of digital platforms. DHIS2’s interpretive flexibility enabled alignment among heterogeneous stakeholders, its structural coherence embedded new practices within established governance routines, and its common identity anchored climate services within familiar health system norms. Through these mechanisms, the platform supported the translation of policy commitments into operational practice and enabled early normalization of climate services within routine health system activities.

By theorizing how digital platforms mediate legitimacy during the establishment of cross-sectoral innovations, this study helps explain why some initiatives gain traction and move toward institutionalization while others falter. For research and practice, the findings underscore the importance of embedding emerging services within trusted digital infrastructures and attending to legitimacy as a socio-technical process that is central to the successful establishment of climate services for health.

## Figures and Tables

**Table 1:** Summary of data sources.

Data source category	Illustrative sources	Analytic contribution
Policy and governance documents	Health National Adaptation Plan; MOH governance guidelines; health−meteorology collaboration framework	Framing of national priorities and formal mandates
Project and system artefacts	Pilot implementation plans and reports; DHIS2 configuration and climate tools	Platform design, technical mediation, and data integration process
Participants observations	Health program meetings; technical working groups and subcommittees; health−meteorology engagement meetings; national stakeholder meetings; platform implementer meetings; DHIS2 configuration sessions; district-level meetings; webinars	Captured legitimacy-seeking practices, governance processes, cross-sector coordination, and early routine use and technical embedding.

**Table 2:** Actors and institutional roles.

Actors	Actor Institutions	Roles
***Seekers***		
Platform implementers	Digital health partner (DHIS2 implementer)	DHIS2 development, configuration, and technical mediation
***Providers/seekers***		
Health system focal points	MOH Environmental health department and health information division	Focal points for climate–health coordination and health data stewardship
***Providers***		
Health program & IT support	MOH Health programs and IT department	Program use, system operations, infrastructure
Sector leaders	Health and meteorological sectors	Policy direction, approval, and oversight
Technical working groups	Climate–Health and health information TWGs	Cross-sector technical guidance and validation
Climate data authorities	Meteorological services department	Climate data provision and custodianship
External partners & researchers	Implementing partners and academia	Funding, technical support, and knowledge production
Sub-national users	District health teams	Routine data use and local implementation

**Table 3:** Summary of legitimacy-building actions and types.

Legitimacy type	Actor actions and platform role	Actors involved
Pragmatic legitimacy	**Action:** *Framed climate services as national priorities; demonstrated feasibility.*	***Seekers:*** Platform implementers; health system focal units
	**Platform role:** *Operationalized policy via dashboards; enabling cross-actor value (interpretive flexibility).*	***Providers:*** Health programs; technical working groups; sector leaders; climate data authorities
	**Action:**Co-created program use cases; demonstrated planning and research value.	***Seekers*:**Health system focal units; platform implementers
	**Platform role:**Enabled tailored analytics in a unified platform; supporting diverse use without fragmentation *(interpretive flexibility, common identity).*	***Providers****:* Health programs; researchers; sub-national users
Moral legitimacy	**Action:** *Secured MOH approval through formal governance processes.* **Platform role:** *Generated standardized outputs; reinforcing trust; institutional fit (structural coherence).*	**Seekers:**Platform implementers **Seekers/Providers:**Health system focal units **Providers**: Health programs; technical working groups; sector leaders
	**Action:** *Established a formal health−meteorology collaboration with defined roles and data sharing.* **Platform role:** *Served as a neutral coordination space; reducing authority contestation (common identity).*	**Seekers:**Platform implementers; health system focal units **Providers:**Sector leaders; Climate data authorities
Emerging cognitive legitimacy	**Action:** *Deployed climate tools in routine DHIS2 workflows and support early use.*	***Seekers*:**Platform implementers; health system focal units
	**Platform role:** *Established new functions into existing periods, hierarchies, and analytics, lowering cognitive barriers (structural coherence).*	***Providers****:* Sub-national users; external partners & researchers; IT support
